# 

**Published:** 1913-07-05

**Authors:** 


### Appointments.

Mr. F. J. Moore has resigned his appointment as third assistant medical officer at Banstead Mental Hospital; and the fourth, fifth, and sixth assistants have been promoted each a grade in the service.

Mr. John Gwynne, M.B., Ch.B., has been appointed second assistant medical officer at the Southwark Infirmary, Dulwich, at a salary of £120 per annum, rising by annual increments of £10 to £140, with emoluments.

The following appointments and reappointments have been confirmed at the Manchester Royal Infirmary :—Appointments.—Mr. Donald E. Core, M.D., Ch.B. (Vict.), M.R.C.P. (Lond.), as assistant medical officer; Mr. S. M. Hattersley, B.A. (Cantab.), M.R.C.S., L.R.C.P., as house physician. Reappointments.—Mr. W. B. Anderton, M.B. (Lond.), M.R.C.S., L.R.C.P., as pathological registrar; Mr. John Gow, M.B., Ch.B. (Vict.), as accident-room house surgeon; Mr. C. E. Lea, M.D. (Vict.), as medical registrar.

Dr. Alfred Matcham, L.R.C.P. (Edin.), M.R.C.S. (Eng.), has resigned his appointments as district medical officer and public vaccinator under the Southwark Board of Guardians. His resignation is to take effect on July 21, on which date Dr. Matcham will have completed forty years' service. He is an old Guy's student, and consequently has spent nearly the whole of his life amongst the poor in the Borough. Mr. E. H. Norman, a member of the Board, spoke of Dr. Matcham's interest in benevolent institutions. He was honorary physician to Miss Sharman's Orphan Homes, West Square, Southwark; surgeon to the Royal South London Dispensary; indeed, there was hardly any benevolent society or institution in which he had not taken a lively interest. His retirement from public service will be a real loss to the poor of Southwark.

### Books Received.

The Scientific Press, Ltd.: "Medical Electricity and Light," by Ettie Sayer, M.B.; "Nursing of Nervous Patients," by E. Ash, M.D.; "Diet in Dyspepsia," by Wm. Tibbles.

Scott, Greenwood, and Son: "Disinfection and Disinfectants," by M. Christian.

H. K. Lewis: "Lewis's Pocket Case Book."

Smith, Elder, and Co.: "How to Diagnose Smallpox," by W. McC. Wanklyn, M.R.C.S.

S. E. Ash and Co., Ltd.: "The Borough and the Borough Hospitals," by R. M. Wingent.

